# Supplementary material for: Redundant microtubule crosslinkers prevent meiotic spindle bending to ensure diploid offspring in C. elegans
Source: PLoS Genet. 2023 Dec 27;19(12):e1011090. doi: 10.1371/journal.pgen.1011090 (PMC10775986; doi:10.1371/journal.pgen.1011090)
Supplement: S1 Table — List of genotypes of all strains used in this paper. (DOCX) [file pgen.1011090.s007.docx]

Table S1

| Strain Name | Genotype Description | Reference |
| --- | --- | --- |
| FM49 | *zyg-8(or484)III; ruIs57 [pie-1p::GFP::tubulin + unc-119(+)] V; itIs37*  *[pie-1p::mCh::H2B::pie-1 3'UTR + unc-119(+)] IV* |  |
| FM125 | *ruIs57 [pie-1p::GFP::tubulin + unc-119(+)] V; itIs37 [pie-1p::mCh::*  *H2B::pie-1 3'UTR + unc-119(+)] IV* |  |
| FM413 | *bmk-1(ok391); ruIs57 [pie-1p::GFP::tubulin + unc-119(+)] V; itIs37*  *[pie-1p::mCh::H2B::pie-1 3'UTR + unc-119(+)] IV* |  |
| FGP36 | *klp-19(fgp2[klp-19::loxP::GFP::FLAG::AID]); ltIs37 [pAA64; pie-1p::*  *mCh::his-58 + unc-119(+)]* | [1] |
| OD4362 | *ltSi1412 [pNA20; Pmex-5::mNeonGreen::tbb-2 operon linker mCh::his-11::Ptbb-2; cb-unc-119(+)]I; unc-119(ed3)III* |  |
| ANA72 | *adeIs1 [mex-5::spd-1::GFP + unc-119(+)] II; unc-119(ed3) III; ltIs37*  *[pie-1p::mCherry::his-58 + unc-119(+)] IV* | [2] |
| FM758 | *klp-19(fgp2[klp-19::loxP::GFP::FLAG::AID]) III; itIs37 [pie-1p::mCh::*  *H2B::pie-1 3'UTR + unc-119(+)] IV; Tir-1 [ Psun-1>TIR1-C1::F2A::*  *mTagBFP2-C1::NLS] II; wjIs76[Cn_unc-119(+); pie-1p::mKate2::*  *tba-2]* |  |
| FM796 | *spd-1(oj5) I; ruIs57[unc-119(+) pie-1::GFP::tubulin] V; itIs37 [pie-1p::mCH::H2B::pie-1 3'UTR + unc-119(+)] IV* |  |
| FM797 | *spd-1(oj5) I; bmk-1 (ok391) V; ruIs57 [pie-1p::GFP::tubulin +*  *unc-119(+)] V; itIs37 [pie-1p::mCh::H2B::pie-1 3'UTR +*  *unc-119(+)] IV* |  |
| FM831 | *ltSi1412 [pNA20; Pmex-5::mNeonGreen::tbb-2 operon linker mCh::his-11::Ptbb-2; cb-unc-119(+)]I; unc-119(ed3)III; dha10[gfp^3xFLAG::zyg-8b]) III* |  |
| FM836 | *zyg-8(dha10[gfp^3xFLAG::zyg-8b]) III; wjIs76[Cn_unc-119(+); pie-1p::*  *mKate2::tba-2]* |  |
| FM837 | *bmk-1(syb5383[bmk-1::3xGAS::GFP]) V; wjIs76[Cn_unc-119(+); pie-1p::*  *mKate2::tba-2]* |  |
| FM895 | *adeIs1 [mex-5::spd-1::GFP + unc-119(+)] II; unc-119(ed3) III; ltIs37*  *[pie-1p::mCherry::his-58 + unc-119(+)] IV; bmk-1 (ok391) V* |  |
| FM898 | *klp-19(fgp2[klp-19::loxP::GFP::FLAG::AID]) III; itIs37 [pie-1p::mCh::*  *H2B::pie-1 3'UTR + unc-119(+)] IV; Tir-1 [ Psun-1>TIR1-C1::F2A::*  *mTagBFP2-C1::NLS] II; bmk-1 (ok391) V; wjIs76[Cn_unc-119(+);*  *pie-1p::mKate2::tba-2]* |  |
| FM948 | *ruIs57[unc-119(+) pie-1::GFP::tubulin] V; itIs37 [pie-1p::mCH::H2B::pie-1 3'UTR + unc-119(+)] IV; bmk-1(syb3914) V* |  |
| FM1015 | *bmk-1(syb5383[bmk-1::3xGAS::GFP]) V; itIs37 [pie-1p::mCh::H2B::pie-1*  *3'UTR + unc-119(+)] IV* |  |
| FM1018 | *bmk-1 (ok391) V; klp-19(fgp2[klp-19::loxP::GFP::FLAG::AID]) III;*  *itIs37 [pie-1p::mCh::H2B::pie-1 3'UTR + unc-119(+)] IV; Tir-1*  *[ Psun-1>TIR1-C1::F2A::mTagBFP2-C1::NLS] II; ruIs57 [pie-1p::GFP::tubulin + unc-119(+)] V* |  |
| FM1032 | *klp-19(fgp2[klp-19::loxP::GFP::FLAG::AID])III; ltIs37 [pAA64; pie-1p::mCh::his-58 + unc-119(+)]; ruIs57 [pie-1p::GFP::tubulin + unc-119(+)] V; Tir-1 [ Psun-1>TIR1-C1::F2A::mTagBFP2-C1::NLS] II* |  |
| FM1040 | *zyg-8(dha10[gfp^3xFLAG::zyg-8b]) III; spd-1(oj5) I; bmk-1 (ok391) V;*  *ruIs57 [pie-1p::GFP::tubulin + unc-119(+)] V; itIs37 [pie-1p::mCh::H2B::pie-1 3'UTR + unc-119(+)] IV* |  |
| FM1124 | *bmk-1 (ok391) V; ltSi1412 [pNA20; Pmex-5::mNeonGreen::tbb-2 operon linker mCh::his-11::Ptbb-2]I; dha10[gfp^3xFLAG::zyg-8b]) III; spd-1(oj5) I* |  |
| FM1161 | *spd-1(oj5) I; bmk-1(syb3914) V; ruIs57 [pie-1p::GFP::tubulin + unc-119(+)] V; itIs37 [pie-1p::mCh::H2B::pie-1 3'UTR + unc-119(+)] IV* |  |
| FM1163 | *zyg-8(dha196[zyg-8::AID]) III; fxIs1 [pie-1p::TIR1::mRuby, I:2851009] I ;  ltIs37 [pAA64; pie-1p::mCh::his-58 + unc-119(+)];ruIs57 [pie-1p::GFP::tubulin + unc-119(+)] V* |  |

**References**

1. Pelisch F, Tammsalu T, Wang B, Jaffray EG, Gartner A, Hay RT. A SUMO-Dependent Protein Network Regulates Chromosome Congression during Oocyte Meiosis. Molecular Cell. 2017; 65(1):66-77. pmid: 27939944.
2. Nahaboo W, Zouak M, Askjaer P, Delattre M. Chromatids segregate without centrosomes during Caenorhabditis elegans mitosis in a Ran- and CLASP-dependent manner. Molecular Biology of the Cell. 2015; 26(11):2020-9. pmid: 25833711.
