# Supplementary material for: Redundant microtubule crosslinkers prevent meiotic spindle bending to ensure diploid offspring in C. elegans
Source: PLoS Genet. 2023 Dec 27;19(12):e1011090. doi: 10.1371/journal.pgen.1011090 (PMC10775986; doi:10.1371/journal.pgen.1011090)
Supplement: Attachment — Submitted filename: Review McNally.docx [file pgen.1011090.s019.docx]

In their current manuscript Li et al explore the roles of a set of MT binding proteins during the late anaphase stage of meiosis I in the nematode *C. elegans*. While these proteins have been studied individually before, the authors uncovered that co-depletion of the proteins SPD-1 or KLP-19, when combined with BMK-1 depletion, led to faster elongation during Anaphase B. Puzzlingly, co-depletion of SPD-1 and KLP-19 led to slower anaphase B. The authors then incorporate the doublecortin-related MT-binding protein ZYG-8 into the story and showed that its depletion slows spindle elongation during anaphase B. They propose that ZYG-8 might achieve this by excluding SDP-1 and KLP-19 from spindles.

Overall, this is an interesting subject for the meiosis and MT communities. I believe the experiments are well performed and the quantitative analysis of the phenotypes makes it easier for the reader to follow. Despite this, I would like to provide my views on specific points for the authors to consider some changes in an attempt to strengthen the link between the presented data and the proposed model(s). My two biggest concerns are 1) the use of the seemingly partial loss-of-function allele *bmk-1(ok391)* as opposed to the full deletion *bmk-1(syb3914)* and 2) the use of GFP::ZYG-8 and *zyg-8(RNAi)* for phenotypic analysis, knowing that GFP::ZYG-8 is in itself a partial loss-of-function and that *zyg-8(RNAi)* leads to partial depletion.

- The authors describe the localisation of both BMK-1 and SPD-1 in the spindle midzone in anaphase. However, their localisation patterns is not . During Anaphase A, while BMK-1 is present in ‘bundles’ along the sides of the chromosomes and exceeding their length, SPD-1 is present in a more tightly packed region, smaller than the length of the chromosomes. Additionally, during anaphase B, while both proteins are in the spindle midzone, BMK-1 fills the whole space between segregating chromosomes whereas SPD-1 is more restricted to the central-most region, with a visible gap between the H2B and SDP-1 signals.
- The authors claim that KLP-19 and SPD-1 localisation are independent of each other (lines 183-186). However, they cite Figure S1E-H for this and neither *klp-19(RNAi)* nor spd-1(RNAi) are tested alone, but rather in the *bmk-1(ok391)* background.

- I wonder why the authors didn’t stick with the complete KO *bmk-1(syb3914)*, for cleaner experiments and an easier interpretation? In this line, if the *bmk-1(syb3914)* allele shows increased velocity, wouldn’t this contradict the statement that ‘…depletion of either KLP-19, SPD-1 or BMK-1 led to normal anaphase B spindle elongation velocity’ (as in the abstract lines 34-35) false? Not considering the technicality of depletion/deletion here.
- In figure S2, when testing double depletions, why is the *bmk-1(syb3914)* allele combined with *spd-1(RNAi)* and the *bmk-1(ok391)* allele combined with the *spd-1(oj5)* allele? The condition *spd-1(RNAi)* alone is missing in the graphs in panels C and D.
- Does localisation of KLP-19 and SPD-1 change in the *bmk-1(syb3914*)?
- Figure 3D is lacking the *bmk-1(ok391)* condition.
- In figure S3, the condition *cyk-4(RNAi)* is missing in the graphs in panels C and E.
- As the authors point out, GFP::ZYG-8 is likely a partial loss-of-function and is therefore not ideal, particularly for quantitative analysis on chromosome segregation or spindle phenotypes. It does appear that the temperature-sensitive *or484* allele could provide a much cleaner (or at the very least, a complementary) approach to assessing phenotypes. The authors analyse the impact of ZYG-8 depletion on SPD-1, KLP-19, and BMK-1 using RNAi, which causes partial depletion, further complicating the analysis, particularly of apparent negative results (like BMK-1).
- In Figure 4, panels A and E show imaging data of lower quality than the rest of the panels in the paper. In A, it also appears the spindle is not in the best angle for imaging, so for a good comparison to other figures, a full-length Metaphase spindle would help. In E, this is more important because an important claim is made regarding ZYG-8 regulating KLP-19. Something that resembles more panel I in Figure S1 would be necessary to have a convincing image along with the graphs.
- Still in Figure 4, including Metaphase and Anaphase A in panels E and G would also help the reader understand whether this increase is specific to anaphase B (same for the other proteins). This would also make it possible to assess where these increases are taking place (midbivalent, chromosomes, spindle).
- The experiment involving CLS-2 depletion is complicated by the fact that if *cls-2(RNAi)* had a fully penetrant effect, there would not be any segregation to measure velocity from. In our experience.
- I like the authors’ models, particularly as a first approach towards understanding the complex, combined action of different MT-binding proteins during oocyte meiosis. However, with the methodology used it is very difficult to assign an anaphase B-specific role for these proteins. Phenotypes could be the consequence of accumulating minor, less noticeable defects in earlier stages of spindle assembly. I don’t think it would be easy to set up AID-system experiments to address this with enough time resolution, so I would suggest mentioning explicitly this limitation in the text, particularly when discussing the models.

Small comments:

- Line 86, did the authors mean ET instead of EM tomography?
- Lines 80-81. Stating that anaphase B is kinetochore independent is an overstatement. While it is true that chromosomes can segregate, they do so in an erroneous way. Clearly there are pushing forces without kt proteins but stating (100%) independence sounds like a strong statement that I am not sure will hold true.
- I wonder why there is so much info on CLS-2 in the Intro (Lines 120-121 and 134-137).
- In line 181, it should read KLP-19::GFP instead of KLP-1::GFP
