# Supplementary material for: Redundant microtubule crosslinkers prevent meiotic spindle bending to ensure diploid offspring in C. elegans
Source: PLoS Genet. 2023 Dec 27;19(12):e1011090. doi: 10.1371/journal.pgen.1011090 (PMC10775986; doi:10.1371/journal.pgen.1011090)
Supplement: Attachment — Submitted filename: Li et al Reviewer Comments_V1.docx [file pgen.1011090.s020.docx]

The presented manuscript seeks to address how *C. elegans* oocyte meiotic spindles, which are comprised of tiled short microtubules, elongate during Anaphase B while maintaining their structural integrity. Microtubule crosslinking, sliding, and polymerization have all been suggested to play roles in the anaphase B spindle, however the specific activities and proteins involved have been unclear. This manuscript describes the overlapping/redundant roles of Kinesin-5/BMK-1, Kinesin-4/KLP-19, and PRC1/SPD-1, as well as the doublecortin-related protein ZYG-8. The authors demonstrate that individual removal of BMK-1, KLP-19, and SPD-1, whether by RNA-interference or loss of function alleles, are insufficient to substantially impact spindle assembly or anaphase chromosome segregation. However, removal of both BMK-1 and either SPD-1 or KLP-19 resulted in fragile hyper-elongated spindles. Conversely, removal of both SPD-1 and KLP-19 slowed anaphase chromosome segregation. Removal of the doublecortin-related protein ZYG-8 resulted in an increase in the recruitment of KLP-19 and SPD-1 to anaphase spindles, which ultimately slowed their rate of separation. Additionally, removal of ZYG-8 also disrupted metaphase spindle morphology and showed a delay in the progression from anaphase A and anaphase B.

As the roles and functions of the different proteins examined here have remained unclear during oocyte meiosis, this manuscript will be of interest and use to the community. However, prior to publication there are specific issues that need clarification as detailed below.

**Major Comments:**

1. Clarifications of methods and conditions are needed for the following:
   a. What conditions were used for the temperature sensitive (TS) alleles? And were the control comparisons carried out at the same temperatures? The methods section does not seem to specify what temperatures were used or how long worms were upshifted to the restrictive temperature prior to imaging.
      i. Lines 339-340, 2 hours at the ‘nonpermissive temperature’ (which is not specified) is quite short – it might be good to mention/cite this paper that used this allele for rapid upshifts during pronuclear centration/rotation that suggests this TS allele is fast acting (PMID 17666432, Bellanger et al 2007)
   b. It is unclear whether/when the authors are looking at spindles during meiosis I or meiosis II or if the data being presented are pooled from both (with the exception of Supplemental Figure S5 which separates the two). To this point, two supplemental videos included (Supplemental Videos 6 and 7) both appear to largely cover meiosis II, but that distinction is not made clear. Ideally these would be replaced with meiosis I videos.
      i. For Supplemental Video S7 showing the ‘broken’ spindle phenotype after *zyg-8* depletion: the broken spindle phenotype also looks somewhat monopolar (or possibly a clustered multipolar), it would be nice to see a spindle pole marker in this condition to better understand this peculiar phenotype.
   c. Whether spindle rotation is normal across the various tested conditions should be reported. Particularly any conditions in which polar body extrusion phenotypes are being discussed as this impacts the orientation of the spindle relative to the ingressing polar body furrows.
2. From the figures presented, some of the polar body extrusion (PBE) phenotypes discussed are unclear as there is no membrane or cortex marker shown and the cytoplasmic background is indistinct. To that end, and similar to point 1C above, it is unclear whether the orientation of the spindle relative to the ingressing polar body extrusion furrows is normal, with furrows ingressing in parallel to the spindle, or if furrow targeting is impaired and ingression is occurring more perpendicular to the spindle. It is also unclear that the spindle bending does precede the myosin-dependent membrane furrows. Visualization of both the spindle and membrane (or myosin) at the same time would help clarify this point to the reader. Finally, the methods section is missing an explanation for the polar body extrusion phenotype distinctions, when they were called, and whether the data presented are specific to meiosis I PBE or meiosis II PBE.
3. Fluorescence intensity quantifications should be normalized to the histone signal to remove any inconsistencies with spindle depth and the depth of the oocyte within the worm uterus across the various perturbations.
4. Figure 5C could use some indication of what the reader should be looking at between the different image panels, as the point being made in the text is unclear from the images. Additional clarification/description in the text may also be helpful. From the images of *zyg-8(or484)* in panel 5C it seems as though the chromosomes are less well aligned/organized in this condition – is this consistent? The text describes the spindle phenotypes after loss of ZYG-8, but does not mention what happens to the organization of the chromosomes within the spindle.
5. Lines 108-112, saying that the exact function of the sorting force during meiosis remains unknown seems a bit disingenuous. The cited reference (22, Cavin-Meza et al 2022) in that section shows that spindles lacking these forces have varying degrees of spindle assembly and chromosome segregation defects.

**Minor Comments:**

1. It is challenging for this reader to switch between the mammalian and *C. elegans* gene/protein names throughout the manuscript while keeping the information being presented straight. Superscripts of the corresponding human orthologue in the text would be potentially helpful here (e.g. SPD-1^PRC1^ for *C. elegans* and PRC1 for human).
2. Numerous figures and diagrams are presented in red/green colors, it would be nice if they could be made more color-blind friendly (cyan-magenta) or information added on how to view a color-blind friendly version of the manuscript (e.g. through Accessibility controls in iOS and equivalent display settings in Windows).
3. Lines 203-206, the mention of GFP::tubulin makes this sentence more confusing than it needs to be.
4. Lines 356-358, would benefit from clarification – as written it sounds like a time/stage specific depletion which it is not. Currently it sounds as if general *zyg-8* depletion is being compared to *zyg-8* depletion/loss of function only in anaphase B.
5. Figure 7 could benefit from simplification, particularly since the spindle/cortex diagram is repeated in all the models. A single central diagram of the meiotic spindle/cortex with the four presented possible models surrounding it would allow for more focus on the different possible models themselves.
6. During the review of this paper another paper from a different group describing live-imaging of KLP-19 depletion was released (PMID 37419936, Pitayu-Nugroho et al 2023). It would be nice if the authors could look over this recent publication and include any similarities or contrasts.
7. Typo on line 181, KLP-1::GFP should be KLP-19::GFP
8. Line 195, depletion should be deletion.
9. Line 511, molted should be molten.

While not required for the revision, there are two outstanding questions presented in the manuscript which could be addressed:

1. Lines 394-397, it is mentioned that testing cortical pulling forces is difficult because of impacts on spindle structure following inhibition of cytoplasmic dynein, but depletion of LIN-5/ASPM-1/CMD-1 have all been shown to prevent spindle rotation (PMID 19219036, van der Voet et al 2009), and *aspm-1* mutant/RNAi oocytes have relatively mild spindle assembly/chromosome segregation defects (PMID 24554763, Connolly et al 2018). While perhaps not perfect, combining aspm-1 inhibition with the conditions analyzed here may provide insight into the spindle bending observed during anaphase.
2. Lines 424-428 discuss how KIF4A inhibits CLASP-driven microtubule polymerization, particularly within microtubule bundles as KIF4A/PRC1 has a higher affinity for bundled microtubules than CLASP does and suggests that KLP-19^KIF4A^ may inhibit CLS-2^CLASP^-dependent anaphase B elongation by a similar mechanism. If this were true, one would expect to see enhanced CLS-2^CLASP^ spindle localization during anaphase B in oocytes lacking KLP-19. Fluorescent-fusions to CLS-2 have been reported previously (PMID 25866924, Maton et al 2015 ; PMID 31243051 Pelisch et al 2019) and may be available from the stock center.
