# Supplementary material for: Redundant microtubule crosslinkers prevent meiotic spindle bending to ensure diploid offspring in C. elegans
Source: PLoS Genet. 2023 Dec 27;19(12):e1011090. doi: 10.1371/journal.pgen.1011090 (PMC10775986; doi:10.1371/journal.pgen.1011090)
Supplement: Attachment — Submitted filename: Response to reviewers.docx [file pgen.1011090.s021.docx]

From the editor: Should you decide to revise the manuscript for further consideration here, your revisions should address the specific points made by each reviewer. In particular, we ask that you include: 1) clarifications of methods and conditions (i.e., whether data is from meiosis I, meiosis II, or both combined);

We have separated meiosis I data from meiosis II data in all figures and added increased n where needed.

2) consider additional strategies to ZYG-8 knockdown given concerns about the use of GFP::ZYG-8 and zyg-8(RNAi) due to their partial loss-of-function and partial depletion, respectively;

We have added zyg-8(RNAi) in a zyg-8(ts) background as well as zyg-8(degron) data. We now present 5 alleles. 3 strong alleles with 100% embryonic lethality and 2 weak alleles with partial lethality. We have not done ALL experiments with all 5 alleles because of the time constraint imposed by the journal.

3) address the issues with the use of bmk-1(ok391) instead of bmk-1(syb3914) raised by reviewers;

Bmk-1(ok391) has been treated as a null allele in multiple publications because of a western blot shown in Bishop et al. (2005) PMID: 15548597. Bmk-1(syb3914) appeared in Cavin-Meza et al. (2022) PMID: 35147496. It was generated by SUNY Biotech for the Wignall lab in a complex strain background: dhc-1(ie28[dhc-1::degron::GFP]) I; EU1067 {unc-119(ed3) III; ruIs32[unc-119(+) pie-1::gfp::h2b] III; ruIs57[unc-119(+) pie-1::GFP::tubulin]}; ieSi38 [sun-1p::TIR1::mRuby::sun-1 3'UTR + Cbr-unc-119(+)] IV; bmk-1(syb3914) V . Cavin-Meza et al. reported results of double and triple depletions with a dynein degron and kl-18(RNAi) and did not report any phenotypic differences between ok391 and syb3914. We obtained the above strain very late in this project and had to do extensive outcrossing to generate strains usable for this project. Because the CRISPR was done by SUNY in a strain with ruIs32, which is a bombarded insertion but appears from crosses to be tightly linked to bmk-1, we cannot make some requested strains with syb3914. We have added syb31914 data to some but not all experiments because of the time involved in making new strains.

and 4) include missing single mutants or depletions in graphs and panels.

We have added single mutants to panels where it is possible without obscuring the data.

We will also require a detailed list of your responses to the review comments and a description of the changes you have made in the manuscript.

See below and changes to the manuscript are in red font.

**Comments to the Authors:
Please note here if the review is uploaded as an attachment.**

Reviewer #1:

However, before these results can be considered acceptable for publication in PLOS Genetics, the authors need to address the following concerns.

Major concerns.

1. The authors conclude that ZYG-8 promotes anaphase B chromosome separation by competing with both SPD-1 and KLP-19 for binding to spindle microtubules. However, the authors only see reduced anaphase B separation rates when using GFP RNAi on a CRISPR generated endogenously tagged GFP::ZYG-8 gene product, and not when using zyg-8 RNAi or zyg-8(or484ts). Furthermore, the authors only show reduced levels of SPD-1 and KLP-19 after RNAi knockdown of untagged endogenous ZYG-8.

As this is a key conclusion of the manuscript, the authors need to (1) demonstrate that an alternative approach to ZYG-8 knockdown also results in reduced anaphase B separation rates. Degron tagging of endogenous zyg-8 is easy to do with CRISPR (if one does not include GFP, and in this case it would be best not to include an FP tag but only the degron tag and assess knockdown effectiveness based on phenotype). Degron tagging generally provides strong knockdowns compared to RNAi, from observations in other studies thus far. The authors also could use zyg-8 RNAi in the zyg-8(or484ts) background at the restrictive temperature.

We have done the suggested additional experiments, zyg-8(RNAi) in the zyg-8(ts) background as well as auxin-induced degradation of ZYG-8. 3/5 alleles for meiosis1 and meiosis 2 now result in 100% embryonic lethality (Fig. S5A) and result in reduced anaphase B velocity (Fig 4G, Fig S5B). 2/5 alleles with partial embryonic lethality (Fig. S5A) do not cause a significant reduction in anaphase B velocity (Fig. 4G, Fig.S5B).

The authors also should (2) show how much GFP RNAi and zyg-8 RNAi knock down the levels of GFP::ZYG-8, and

GFP::ZYG-8 is dim, making it difficult to assay statistically significant differences between partial depletions. We feel that showing the 3 alleles with the strongest lethality result in the slowest anaphase B addresses this overall issue.

(3) if zyg-8 RNAi reduces the already low levels of GFP::ZYG-8 to background, the authors should assess KLP-19 and SPD-1 levels after GFP RNAi in the GFP::ZYG-8 background with the other proteins also GFP tagged. Presumably one would see a greater reduction after GFP RNAi than by zyg-8 RNAi alone, given the difference in phenotypes seen with the two different approaches to reducing zyg-8 function.

We agree with the reviewers logic. We now show that the weak depletion, zyg-8(RNAi) affects GFP::spd-1 intensity but not GFP::tubulin intensity. The stronger depletions affect GFP::tubulin intensity which makes interpretation of GFP::SPD-1 intensity less interpretable.

These are important experiments to do given the unlikely but real possibility that GFP tagging of ZYG-8 somehow alters its function in a neomorphic fashion, as the authors have only observed a requirement for anaphase using this GFP tagged allele.

We have added the requested degron data and RNAi on top of ts data and hope this satisfies the reviewers concerns.

2. Also with respect to ZYG-8, the authors state in lines 350-353 that reducing zyg-8 function (either with or484ts or via GFP RNAi in the GFP:ZYG-8 background) reduced anaphase A velocity, as documented in Fig S4G. In line 358, the authors also conclude that chromosome separation rate decreases after zyg-8 depletion, using the zyg-8 allele or484ts and specifying anaphase B in the text. However, Fig 5E shows increased velocity during anaphase A for zyg-8(or484) compared to control, and the difference appears significant (and certainly not decreased). The authors need to address these contradictory results (S4G vs 5E). Also, in S4G it appears that or484ts reduces anaphase A velocity as much or more than GFP RNAi, which is in contrast to the stronger effect with GFP RNAi for reducing anaphase B velocity, further highlighting the need to more conclusively examine ZYG-8 knockdown using a degron tag.
These contradictions are due to the nature of the zyg-8 phenotype and the time periods chosen for measuring anaphase A velocities (0-75 seconds before anaphase B onset). A major phenotype of zyg-8 depletion is the introduction of a pause period between anaphase A and anaphase B. This pause is obvious in previous Fig. 5E. The slower mean velocity in S4G included the pause period, because the objective choice for the time of anaphase A is the time between initiation of homolog separation and the initiation of spindle elongation, shown in current Fig 7A and C.

3. The authors combine data from both meiosis I and II, and yet there are substantial differences in oocyte meiotic spindle assembly during meiosis I and II, and moreover very little is known about meiosis II requirements given the earlier defects seen during meiosis I when analyzing gene functions. It is telling that the authors never even mention in the text that they are combining these data but mention it only in the Methods and never even refer to the supplemental figure showing the meiosis I and II comparisons except in the Methods! The authors should have been more transparent and should have indicated that they were combing meiosis I and II data within the Results section. Furthermore, in the supplemental figure showing the comparisons (S5), some of the conditions are quantified in only 2 or 3 or 4 oocytes, making the lack of statistical significance unreliable. It is confusing and creates a major caveat to combine data from meiosis I and II; the authors should provide only data for meiosis 1, or separate the data for meiosis I and II and provide sufficient examples to make the statistical analyses rigorous throughout.
We have separated meiosis I data from meiosis II data and added additional measurements to increase statistical rigor.

4. The authors use the Student’s T test throughout the manuscript. Such a test is appropriate when the data show a normal distribution, which these kind of biological data usually do not show. The authors should either document normal distributions or preferably use the more rigorous U-test to better assess the significance of the differences they document in their comparisons.

We have recalculated all p values using the Mann-Whitney U test for non-parametric data using Prism.

5. In lines 267-269, the authors set out to test whether SPD-1 works in a complex with CYK-4 by depleting CYK-4 in the bmk-1(ok391) background. Because spd-1;bmk-1 double mutants show increased velocity, and the cyk-4;bmk-1 double mutant does not, they conclude the SPD-1 and CYK-4 do not act in a complex. This is reasonable logic, although the authors should state it more explicitly (they refer the lack of phenotype with cyk-4; bmk-1 and not how it differs from spd-1; bmk-1 double mutants). However, to be more conclusive, the authors should use the syb3914 allele of bmk-1, which they document is stronger than ok391. In general, it is not clear why the authors often use bmk-1(391) instead of syb3914, given that syb3914 showed increased anaphase B velocity while ok391 did not. In this case, to make the negative result more compelling, the authors should use the stronger bmk-1 allele syb3914. In addition, the authors should report the anaphase velocity when both CYK-4 and SPD-1 are reduced, as it is possible that a cyk-4; spd-1 double mutant might exhibit increased velocity if they function independently in this setting to stabilize the central spindle.

We have added text to the results indicating a significant difference in anaphase B velocity between bmk-1(ok391); spd-1(RNAi) vs bmk-1(ok391); cyk-4(RNAi). We agree that the additional double mutants suggested by the reviewer would indeed be interesting. However, this requires new strain construction and we did not have time for this within the time constraints set by the journal.

Minor comments.

1.In Lines 163-164 the authors claim that BMK-1 does not localize to the poles during anaphase. However there does seem to be GFP:BMK-1 signal on the pole side of the chromosomes during anaphase A, both in Figure 1B and even more so in S1A where there is some yellow in the merge that is even centrally positioned.
We have changed the sentence to: “At anaphase A, BMK-1::GFP localized strongly at the midzone of the spindle but its intensity at the two poles of the shortening spindle was much lower relative to the mKate::tubulin signal (Fig. S1A).” to more accurately describe the data.

2. The authors use two alleles of bmk-1, the partial deletion ok391 and the complete deletion syb3914, and the authors see an increase in anaphase B separate rate only with syb3914. However, in S2C the authors document a greater difference in anaphase B velocity when comparing ok391 with ok391;oj5, than they do when comparing syb3914 and syb3914; spd-1 RNAi. It would be helpful if the authors could document more consistency (in syb-3914 being stronger than ok391) by comparing syb3914 to syb3914;oj5, as they do with ok391.
bmk-1(sb3914); spd-1(oj5) double mutant data has been added to Figs 1I, S1E, 2F and 3E.

3. The authors often leave out single mutants for their graphs showing velocity: S1E lacks klp-19 single mutant; Fig 2D only shows control and double mutant with neither single mutant; S2D does not show spd-1 RNAi or bmk-1(ok391) single mutants; Fig 3C lacks bmk-1(ok391) single mutant; 3E lacks bmk-1(ok391) and cyk-4 RNAi single mutants. It would be better to more consistently show all relevant single mutant genotypes.
Single mutants have been added to Fig. S1E. The missing single mutants in Fig. 2D are shown in Fig. 1I. We have added two single mutants to Fig. 2D but the format of Fig. 2D does not allow adding more genotypes without obscuring the data. The format of Fig. 1I allows inclusion of larger numbers of genotypes. We have added *bmk-1(ok391) and spd-1(RNAi)* single mutant data to Fig. 2D.

4. In Figure 2, the authors distinguish between polar body not extruded, and polar body retracted. However, the live imaging frame examples shown for each polar body phenotype shows no difference between these two. Presumably one would have to show an early and late time point to document retraction. It is not clear that this difference matters (how long they did wait before deciding it didn’t retract; maybe some retracted later, etc.). It would be simpler to just call them all failed extrusion.

We have combined polar body not extruded and polar body retracted into one category called polar body extrusion failure to Fig 2F, S3E, 3D and S4F.

5. In Fig 5G, the authors quantify the time from the beginning of spindle rotation to the beginning of anaphase B. Does some spindle rotation occur during metaphase? Would it be better to simply quantify the time from the beginning of anaphase A (first time point when increased separation is detected) to beginning of anaphase (first time point when poles move apart)?
In control time-lapse sequences, 3 APC-dependent events always occur sequentially (1. initiation of spindle shortening, 2. rotation, 3. Homolog separation). The anaphase A phenotype of zyg-8 depletion is complex and we are trying to report it as clearly as possible. Homolog separation occur before rotation in zyg-8. We have moved relevant data to Fig. 6.

6. Some typos: line 181, which should read KLP-19:GFP; Corrected.

line 203 change Fig 1J-K to Fig 1K; line 271, change Fig S3C-D to Fig S3D; line 353 change S4F to S4G;

Because we have re-arranged and added figures, we have checked figure references throughout the manuscript.

Reviewer ?

However, prior to publication there are specific issues that need clarification as detailed below.

**Major Comments:**

1. Clarifications of methods and conditions are needed for the following:
   a. What conditions were used for the temperature sensitive (TS) alleles? And were the control comparisons carried out at the same temperatures? The methods section does not seem to specify what temperatures were used or how long worms were upshifted to the restrictive temperature prior to imaging.

Temperature sensitive alleles are filmed after 1.5 hours of incubation at restrictive temperature (25°C). We confirmed the efficiency of the temperature shift for this TS allele by the rectangular or broken metaphase spindle phenotype observed in other methods of ZYG-8 depletion.

i. Lines 339-340, 2 hours at the ‘nonpermissive temperature’ (which is not specified) is quite short – it might be good to mention/cite this paper that used this allele for rapid upshifts during pronuclear centration/rotation that suggests this TS allele is fast acting (PMID 17666432, Bellanger et al 2007)

We have added the ts information and cited the corresponding paper in the materials and methods section.

b. It is unclear whether/when the authors are looking at spindles during meiosis I or meiosis II or if the data being presented are pooled from both (with the exception of Supplemental Figure S5 which separates the two). To this point, two supplemental videos included (Supplemental Videos 6 and 7) both appear to largely cover meiosis II, but that distinction is not made clear. Ideally these would be replaced with meiosis I videos.

Meiosis I data and meiosis II data have been separated in the revised manuscript.

i. For Supplemental Video S7 showing the ‘broken’ spindle phenotype after *zyg-8* depletion: the broken spindle phenotype also looks somewhat monopolar (or possibly a clustered multipolar), it would be nice to see a spindle pole marker in this condition to better understand this peculiar phenotype.

We agree that this would be an interesting addition. However, this requires new strain construction and we did not have time for this within the time constraints set by the journal.

c. Whether spindle rotation is normal across the various tested conditions should be reported. Particularly any conditions in which polar body extrusion phenotypes are being discussed as this impacts the orientation of the spindle relative to the ingressing polar body furrows.

We have not observed any spindle rotation defect in the *bmk-1, spd-1 and klp-19* single or double mutants. We have added the corresponding information to the manuscript.

2. From the figures presented, some of the polar body extrusion (PBE) phenotypes discussed are unclear as there is no membrane or cortex marker shown and the cytoplasmic background is indistinct. To that end, and similar to point 1C above, it is unclear whether the orientation of the spindle relative to the ingressing polar body extrusion furrows is normal, with furrows ingressing in parallel to the spindle, or if furrow targeting is impaired and ingression is occurring more perpendicular to the spindle. It is also unclear that the spindle bending does precede the myosin-dependent membrane furrows. Visualization of both the spindle and membrane (or myosin) at the same time would help clarify this point to the reader. Finally, the methods section is missing an explanation for the polar body extrusion phenotype distinctions, when they were called, and whether the data presented are specific to meiosis I PBE or meiosis II PBE.

We have added the sentence: “These defects were not an indirect consequence of spindle rotation defects as all anaphase spindles started perpendicular to the cortex before spindle bending.” And included separate bar graphs for meiosis I and II.

3. Fluorescence intensity quantifications should be normalized to the histone signal to remove any inconsistencies with spindle depth and the depth of the oocyte within the worm uterus across the various perturbations.

All fluorescence intensities in the revised manuscript are ratios to mCherry::histone intensity.

4. Figure 5C could use some indication of what the reader should be looking at between the different image panels, as the point being made in the text is unclear from the images. Additional clarification/description in the text may also be helpful. From the images of *zyg-8(or484)* in panel 5C it seems as though the chromosomes are less well aligned/organized in this condition – is this consistent? The text describes the spindle phenotypes after loss of ZYG-8, but does not mention what happens to the organization of the chromosomes within the spindle.

We have changed the representative images of zyg-8(or484) to better show the pause in chromosome separation. Definitively scoring congression defects would require z-stacks and all of our live imaging is single focal plane imaging. We cannot make a definitive conclusion about congression so this is not described in the results.

5. Lines 108-112, saying that the exact function of the sorting force during meiosis remains unknown seems a bit disingenuous. The cited reference (22, Cavin-Meza et al 2022) in that section shows that spindles lacking these forces have varying degrees of spindle assembly and chromosome segregation defects.

We have deleted the offending sentence.

**Minor Comments:**

1. It is challenging for this reader to switch between the mammalian and *C. elegans* gene/protein names throughout the manuscript while keeping the information being presented straight. Superscripts of the corresponding human orthologue in the text would be potentially helpful here (e.g. SPD-1^PRC1^ for *C. elegans* and PRC1 for human).

We have added the requested superscripts for proteins in the text but not for specific alleles, specific fluorescent protein fusion, or other instances that we do not want to imply things about homologs outside of *C. elegans*. We have not added the superscripts to the figures or figure legends.

2. Numerous figures and diagrams are presented in red/green colors, it would be nice if they could be made more color-blind friendly (cyan-magenta) or information added on how to view a color-blind friendly version of the manuscript (e.g. through Accessibility controls in iOS and equivalent display settings in Windows).

Figures have been changed to color blind friendly colors.

3. Lines 203-206, the mention of GFP::tubulin makes this sentence more confusing than it needs to be. We have deleted “without GFP::tubulin” from the sentence.
4. Lines 356-358, would benefit from clarification – as written it sounds like a time/stage specific depletion which it is not. Currently it sounds as if general *zyg-8* depletion is being compared to *zyg-8* depletion/loss of function only in anaphase B. We have deleted the first phrase of this sentence which seemed to be responsible for the confusion.
5. Figure 7 could benefit from simplification, particularly since the spindle/cortex diagram is repeated in all the models. A single central diagram of the meiotic spindle/cortex with the four presented possible models surrounding it would allow for more focus on the different possible models themselves.

We have deleted the spindle/cortex diagram to simplfy the figure.

6. During the review of this paper another paper from a different group describing live-imaging of KLP-19 depletion was released (PMID 37419936, Pitayu-Nugroho et al 2023). It would be nice if the authors could look over this recent publication and include any similarities or contrasts. Pitayu-Nugrohu found no defects in single depletions of KLP-19 and we now cite this result.
7. Typo on line 181, KLP-1::GFP should be KLP-19::GFP

Corrected.

8. Line 195, depletion should be deletion.

Corrected.

9. Line 511, molted should be molten. Corrected.

While not required for the revision, there are two outstanding questions presented in the manuscript which could be addressed:

1. Lines 394-397, it is mentioned that testing cortical pulling forces is difficult because of impacts on spindle structure following inhibition of cytoplasmic dynein, but depletion of LIN-5/ASPM-1/CMD-1 have all been shown to prevent spindle rotation (PMID 19219036, van der Voet et al 2009), and *aspm-1* mutant/RNAi oocytes have relatively mild spindle assembly/chromosome segregation defects (PMID 24554763, Connolly et al 2018). While perhaps not perfect, combining aspm-1 inhibition with the conditions analyzed here may provide insight into the spindle bending observed during anaphase.

We agree that it would be nice to specifically remove cortical pulling. In mitosis, this is done with gpr-1,2(RNAi) which does not affect meiotic spindle rotation in our hands. In our hands, aspm-1(RNAi) causes the same metaphase spindle defects as dhc-1(RNAi or degron).

2. Lines 424-428 discuss how KIF4A inhibits CLASP-driven microtubule polymerization, particularly within microtubule bundles as KIF4A/PRC1 has a higher affinity for bundled microtubules than CLASP does and suggests that KLP-19^KIF4A^ may inhibit CLS-2^CLASP^-dependent anaphase B elongation by a similar mechanism. If this were true, one would expect to see enhanced CLS-2^CLASP^ spindle localization during anaphase B in oocytes lacking KLP-19. Fluorescent-fusions to CLS-2 have been reported previously (PMID 25866924, Maton et al 2015 ; PMID 31243051 Pelisch et al 2019) and may be available from the stock center.

We did klp-19(RNAi) on GFP::CLS-2 and saw no significant difference. Because this is RNAi, we think this negative result is a weak result that should not be included. Doing it with the klp-19 degron would be stronger but this would require new strain construction and we did not have time for this within the time constraints set by the journal.

Reviewer ?

Despite this, I would like to provide my views on specific points for the authors to consider some changes in an attempt to strengthen the link between the presented data and the proposed model(s). My two biggest concerns are 1) the use of the seemingly partial loss-of-function allele *bmk-1(ok391)* as opposed to the full deletion *bmk-1(syb3914)* and 2) the use of GFP::ZYG-8 and *zyg-8(RNAi)* for phenotypic analysis, knowing that GFP::ZYG-8 is in itself a partial loss-of-function and that *zyg-8(RNAi)* leads to partial depletion.

- The authors describe the localisation of both BMK-1 and SPD-1 in the spindle midzone in anaphase. However, their localisation patterns is not . During Anaphase A, while BMK-1 is present in ‘bundles’ along the sides of the chromosomes and exceeding their length, SPD-1 is present in a more tightly packed region, smaller than the length of the chromosomes. Additionally, during anaphase B, while both proteins are in the spindle midzone, BMK-1 fills the whole space between segregating chromosomes whereas SPD-1 is more restricted to the central-most region, with a visible gap between the H2B and SDP-1 signals.

We infer that the reviewer meant that “their localization patterns are not identical”. We do not state that the patterns are identical. Without making a BMK-1::GFP, SPD-1::mCherry strain, we would not be comfortable making statements about differences between their localization.

- The authors claim that KLP-19 and SPD-1 localisation are independent of each other (lines 183-186). However, they cite Figure S1E-H for this and neither *klp-19(RNAi)* nor spd-1(RNAi) are tested alone, but rather in the *bmk-1(ok391)* background.

We have deleted “independently of each other” from the subtitle. We have changed the results text to match the data shown.

- I wonder why the authors didn’t stick with the complete KO *bmk-1(syb3914)*, for cleaner experiments and an easier interpretation? This is addressed above in response to the editors queries. In this line, if the *bmk-1(syb3914)* allele shows increased velocity, wouldn’t this contradict the statement that ‘…depletion of either KLP-19, SPD-1 or BMK-1 led to normal anaphase B spindle elongation velocity’ (as in the abstract lines 34-35) false? Not considering the technicality of depletion/deletion here.

In the abstract we have changed “normal” to “had minimal effects”. We have made a similar change to the results text.

- In figure S2, when testing double depletions, why is the *bmk-1(syb3914)* allele combined with *spd-1(RNAi)* and the *bmk-1(ok391)* allele combined with the *spd-1(oj5)* allele? The condition *spd-1(RNAi)* alone is missing in the graphs in panels C and D.

Additional single and double mutant measurements have been added to Fig. 1I.

- Does localisation of KLP-19 and SPD-1 change in the *bmk-1(syb3914*)?

As described above, syb3914 was made by CRISPR in a strain with a tightly linked GFP::tubulin transgene.

- Figure 3D is lacking the *bmk-1(ok391)* condition. Corrected.
- In figure S3, the condition *cyk-4(RNAi)* is missing in the graphs in panels C and E. Corrected.
- As the authors point out, GFP::ZYG-8 is likely a partial loss-of-function and is therefore not ideal, particularly for quantitative analysis on chromosome segregation or spindle phenotypes. It does appear that the temperature-sensitive *or484* allele could provide a much cleaner (or at the very least, a complementary) approach to assessing phenotypes. The authors analyse the impact of ZYG-8 depletion on SPD-1, KLP-19, and BMK-1 using RNAi, which causes partial depletion, further complicating the analysis, particularly of apparent negative results (like BMK-1).

We have added data from a ZYG-8 degron and from zyg-8(RNAi) in the zyg-8(ts) background.

- In Figure 4, panels A and E show imaging data of lower quality than the rest of the panels in the paper. In A, it also appears the spindle is not in the best angle for imaging, so for a good comparison to other figures, a full-length Metaphase spindle would help. In E, this is more important because an important claim is made regarding ZYG-8 regulating KLP-19. Something that resembles more panel I in Figure S1 would be necessary to have a convincing image along with the graphs.

We added images of metaphase spindles to show the effect of ZYG-8 depletion on KLP-19 signals. All fluorescence intensities in the revised manuscript are ratios to mCherry::histone intensity, and we separated results from meiosis 1 vs. meiosis 2.

- Still in Figure 4, including Metaphase and Anaphase A in panels E and G would also help the reader understand whether this increase is specific to anaphase B (same for the other proteins). This would also make it possible to assess where these increases are taking place (midbivalent, chromosomes, spindle).

We added images of metaphase spindles for both conditions. Quantifications of KLP-19 signal intensity during metaphase were also added to the corresponding figure.

- The experiment involving CLS-2 depletion is complicated by the fact that if *cls-2(RNAi)* had a fully penetrant effect, there would not be any segregation to measure velocity from. In our experience.

We deleted this result.

- I like the authors’ models, particularly as a first approach towards understanding the complex, combined action of different MT-binding proteins during oocyte meiosis. However, with the methodology used it is very difficult to assign an anaphase B-specific role for these proteins. Phenotypes could be the consequence of accumulating minor, less noticeable defects in earlier stages of spindle assembly. I don’t think it would be easy to set up AID-system experiments to address this with enough time resolution, so I would suggest mentioning explicitly this limitation in the text, particularly when discussing the models.

We have added :”Metaphase spindle structure and anaphase A velocities appeared normal in single or double depletions of BMK-1^Eg5^, SPD-1^PRC1^, or KLP-19^KIF4A^ (Fig. 7A, Fig. S6A). However, it remains possible that subtle defects early in the cell cycle are responsible for the anaphase B phenotypes observed. “

Small comments:

- Line 86, did the authors mean ET instead of EM tomography? Corrected.
- Lines 80-81. Stating that anaphase B is kinetochore independent is an overstatement. While it is true that chromosomes can segregate, they do so in an erroneous way. Clearly there are pushing forces without kt proteins but stating (100%) independence sounds like a strong statement that I am not sure will hold true. We have deleted “kinetochore independent” from the offending sentence.
- I wonder why there is so much info on CLS-2 in the Intro (Lines 120-121 and 134-137).
- CLS-2 is a candidate for a major factor driving anaphase spindle elongation.
- In line 181, it should read KLP-19::GFP instead of KLP-1::GFP . Corrected.
